# Author Correction: River interlinking alters land-atmosphere feedback and changes the Indian summer monsoon

**DOI:** 10.1038/s41467-024-44829-8

**Published:** 2024-01-11

**Authors:** Tejasvi Chauhan, Anjana Devanand, Mathew Koll Roxy, Karumuri Ashok, Subimal Ghosh

**Affiliations:** 1 Department of Civil Engineering, Indian Institute of Technology Bombay, Mumbai, India; 2 Interdisciplinary Program in Climate Studies, Indian Institute of Technology Bombay, Mumbai, India; 3 Australian Research Council Centre of Excellence for Climate Extremes, University of New South Wales, Sydney, NSW Australia; 4 Climate Change Research Centre, University of New South Wales, Sydney, NSW Australia; 5 Centre for Climate Change Research, Indian Institute of Tropical Meteorology, Ministry of Earth Sciences, Pune, India; 6 Centre for Earth, Ocean and Atmospheric Sciences, University of Hyderabad, Hyderabad, India; 7 Physical Science and Engineering, King Abdullah University of Science and Technology, Thuwal, Saudi Arabia

**Keywords:** Climate sciences, Hydrology, Environmental sciences

Correction to: *Nature Communications* 10.1038/s41467-023-41668-x, published online 22 September 2023

The original version of this Article contained an error in Equation (3), where a minus sign was missing and incorrectly read:

$${{{\rm{H}}}}({{{{\rm{X}}}}}_{{{{\rm{t}}}}})=\mathop{\sum }\limits_{{{{\rm{i}}}}=1}^{{{{\rm{m}}}}}{{{{\rm{p}}}}}_{{{{\rm{i}}}}}({{{{\rm{x}}}}}_{{{{\rm{t}}}}})(\log ({{{{\rm{p}}}}}_{{{{\rm{i}}}}}({{{{\rm{x}}}}}_{{{{\rm{t}}}}})))$$

The correct form of Equation (3) is:

$${{{\rm{H}}}}({{{{\rm{X}}}}}_{{{{\rm{t}}}}})=-\mathop{\sum }\limits_{{{{\rm{i}}}}=1}^{{{{\rm{m}}}}}{{{{\rm{p}}}}}_{{{{\rm{i}}}}}({{{{\rm{x}}}}}_{{{{\rm{t}}}}})(\log ({{{{\rm{p}}}}}_{{{{\rm{i}}}}}({{{{\rm{x}}}}}_{{{{\rm{t}}}}})))$$

This has been corrected in the PDF and HTML versions of the Article.

