# Supplementary material for: River interlinking alters land-atmosphere feedback and changes the Indian summer monsoon
Source: Nat Commun. 2023 Sep 22;14:5928. doi: 10.1038/s41467-023-41668-x (PMC10517128; doi:10.1038/s41467-023-41668-x)
Supplement: Supplementary file 1 — Supplementary Information [file 41467_2023_41668_MOESM1_ESM.pdf]

Supplementary Information for

River interlinking alters land-atmosphere feedback and changes the Indian summer monsoon.

Tejasvi Chauhan[1], Anjana Devanand[2,3,4], Mathew Koll Roxy[5], Karumuri Ashok [6,7], Subimal Ghosh[1,2,*]

1. Department of Civil Engineering, Indian Institute of Technology Bombay, Mumbai, India
2. Interdisciplinary Program in Climate Studies, Indian Institute of Technology Bombay, Mumbai, India
3. Australian Research Council Centre of Excellence for Climate Extremes, University of New South Wales, Sydney, NSW, Australia
4. Climate Change Research Centre, University of New South Wales, Sydney, NSW, Australia
5. Centre for Climate Change Research, Indian Institute of Tropical Meteorology, Ministry of Earth Science, Pune, India
6. Centre for Earth, Ocean and Atmospheric Sciences, University of Hyderabad, Hyderabad, India.
7. Physical Science and Engineering, King Abdullah University of Science and Technology, Saudi Arabia.

[*]Corresponding Author. Email: subimal@iitb.ac.in

Contents

7 Supplementary Figures

3 Supplementary Tables

Supplementary Figures

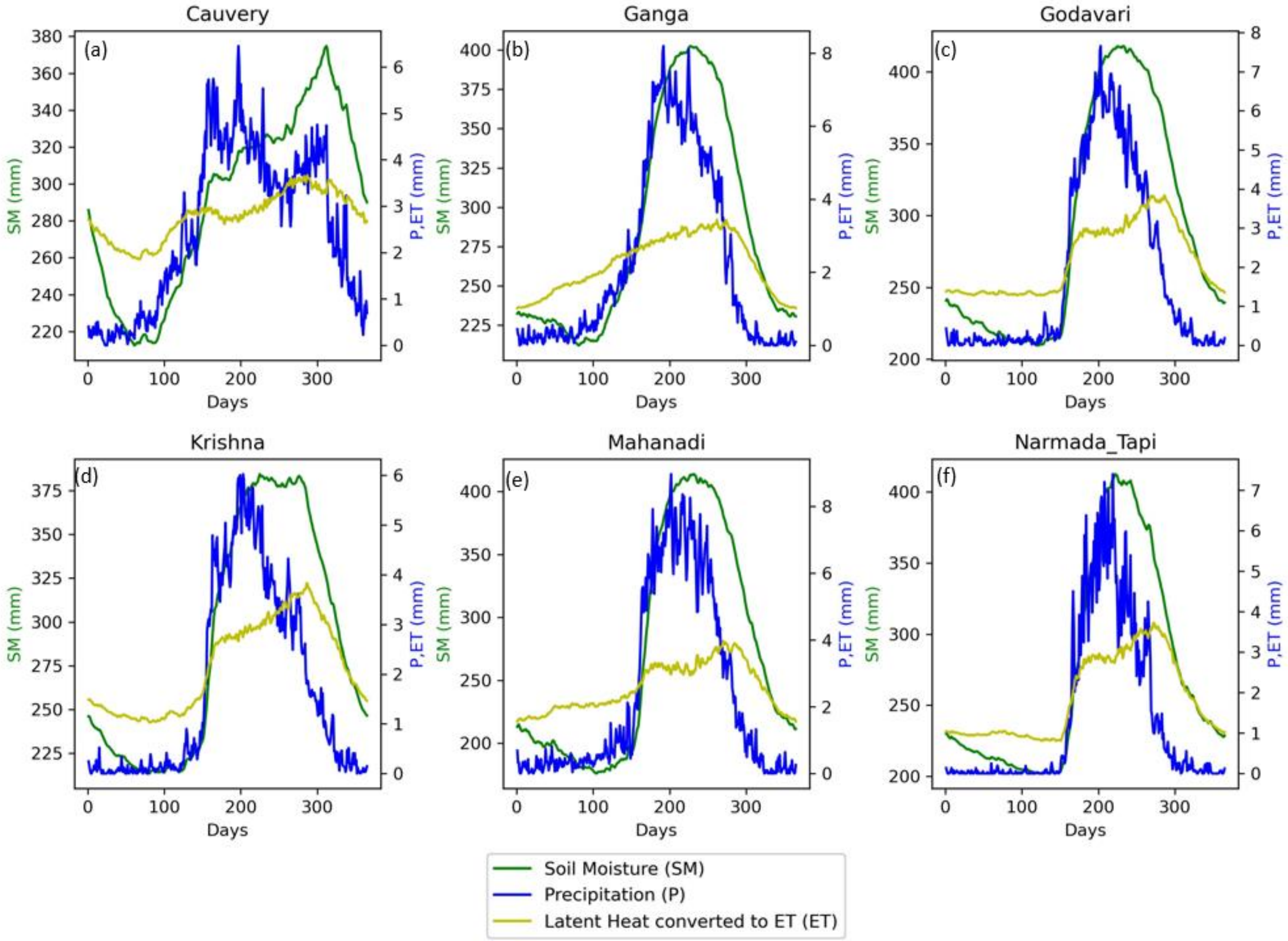

S1: Climatology of soil moisture (SM), precipitation (P), and evapotranspiration (ET) in Cauvery (a), Ganga (b), Godavari (c), Krishna (d), Mahanadi (e), and Narmada and Tapi (f) basins, from the ERA-5 reanalysis.

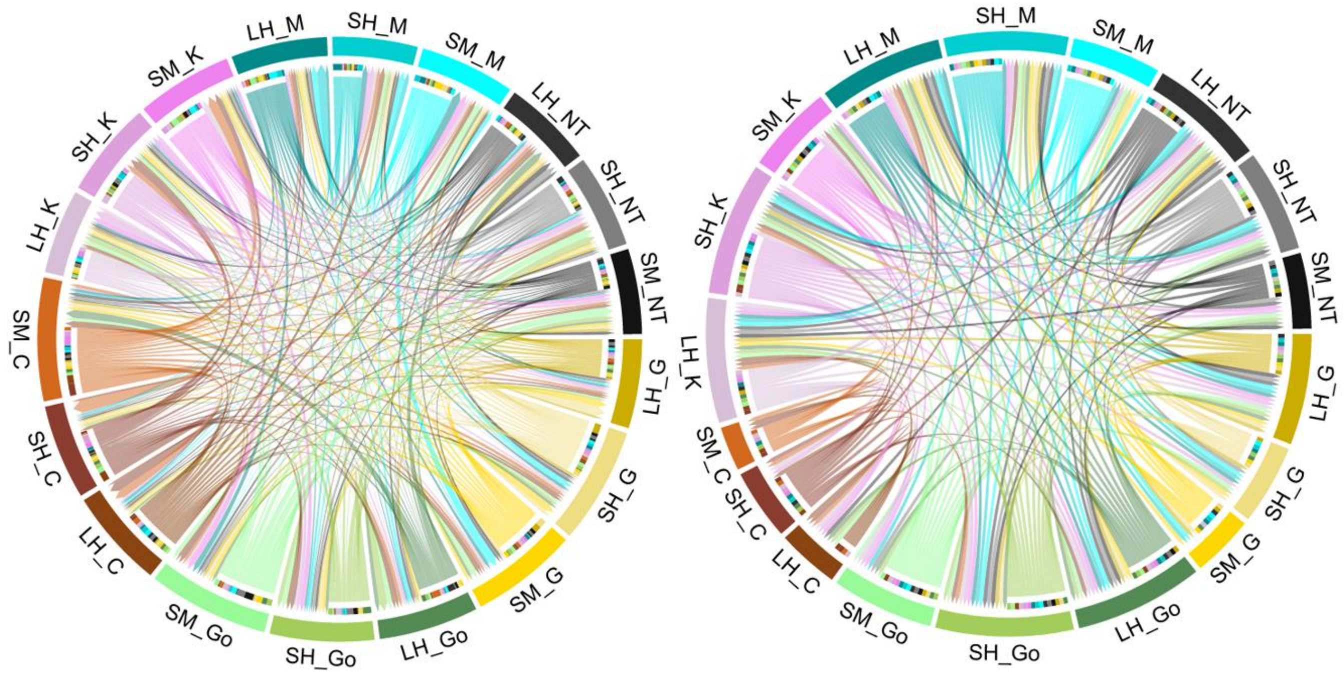

S2: Land-Land connections using Granger Causality (GC, a) and Transfer Entropy (TE, b). Links are only shown if found statistically significant at 99% confidence level.

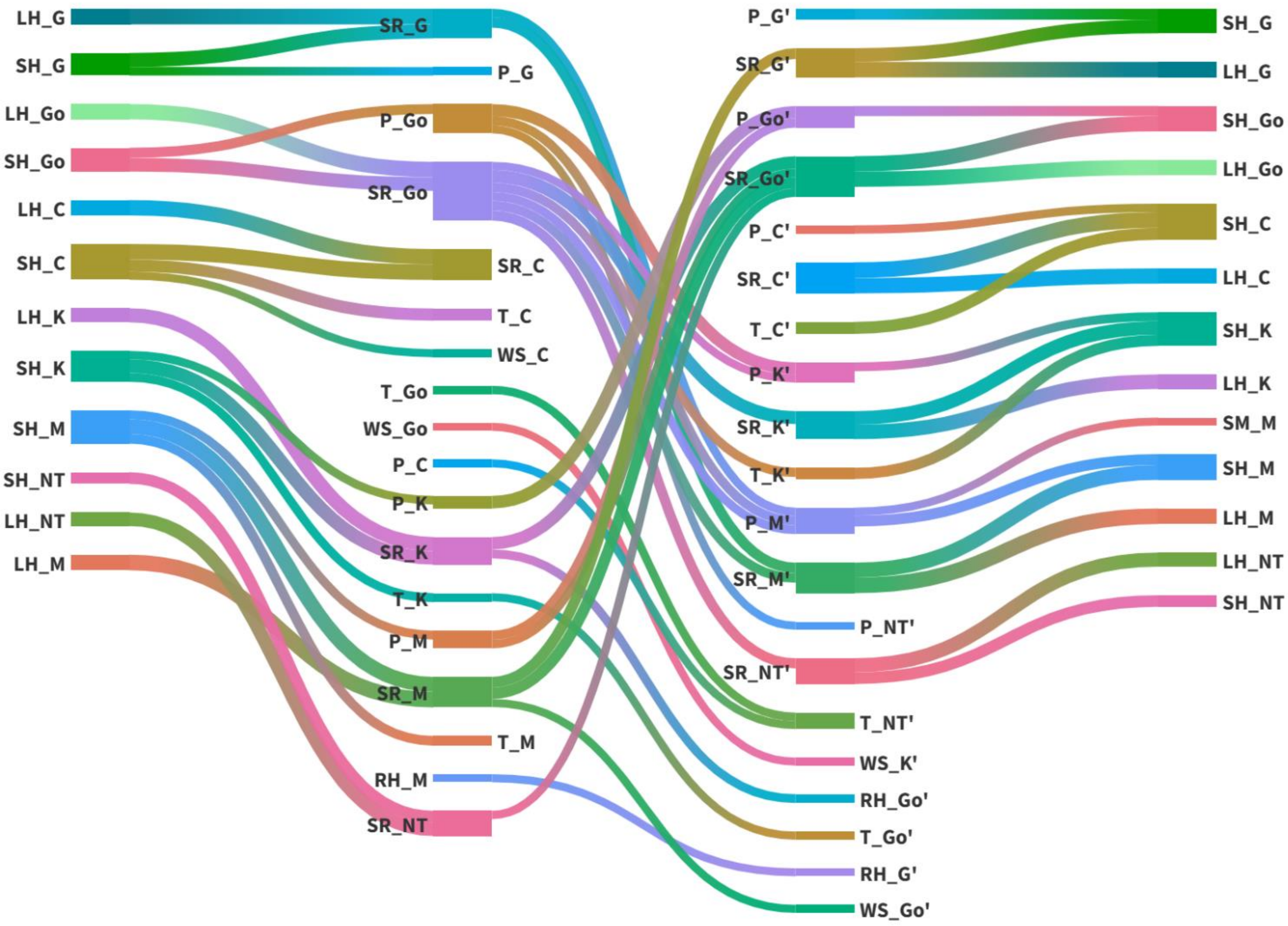

S3: Inter-basin connections via land-atmosphere feedback. Causal connections of land atmosphere interactions from MERRA-2 Reanalysis using PCMCI (similar to Figure 2). A link is shown only if it is found statistically significant at 5% significance level ($p<0.05$) more than 50% of the time (20 years out of 40 years (1981-2020)).

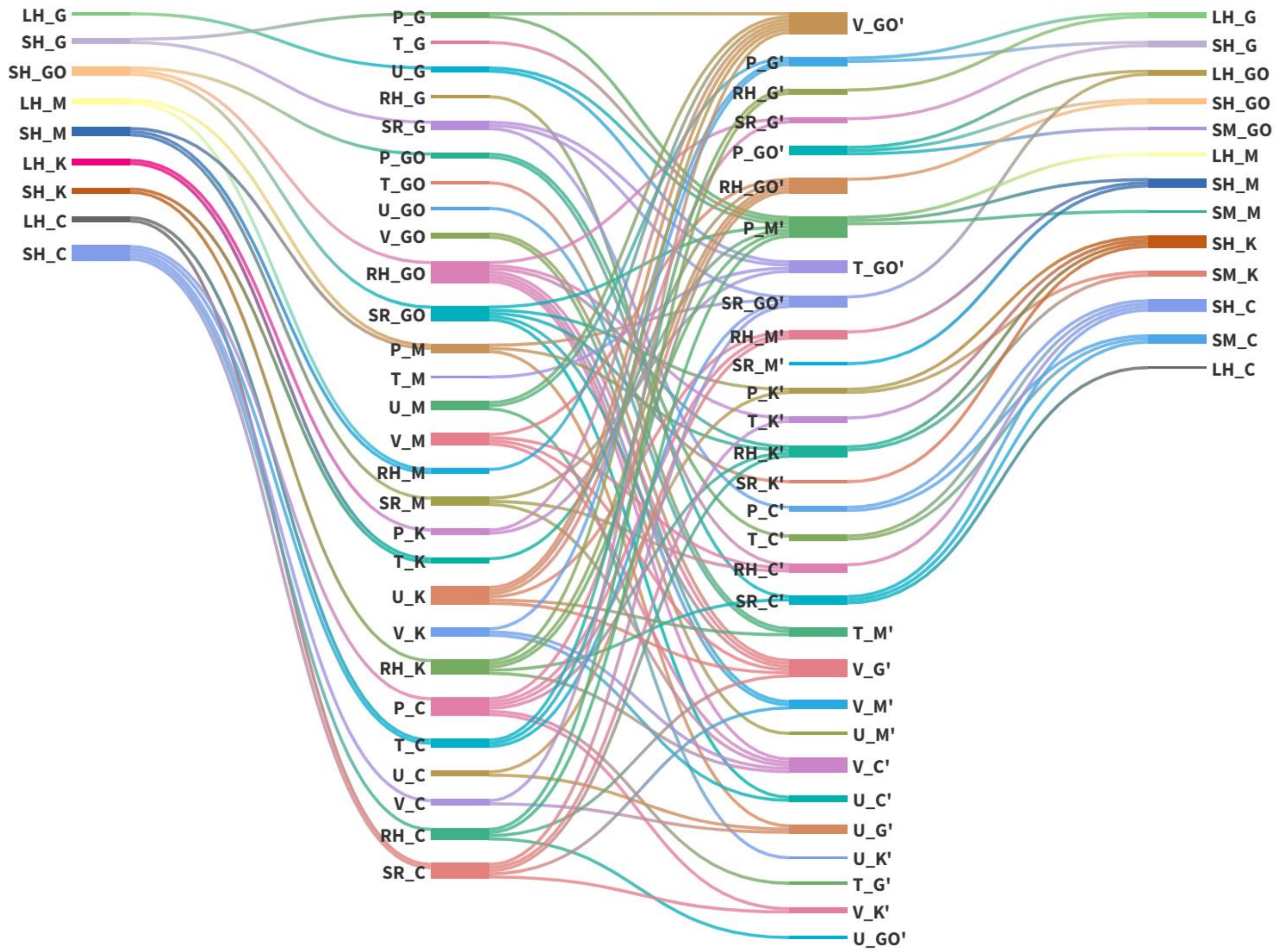

S4: Causal connections of land atmosphere interactions across different basins in control run (CTL) simulations in the regional climate model (WRF-CLM4) using PCMCI (similar to Figure 2). A link is shown only if it is found statistically significant at 5% significant level ($p<0.05$) more than 50% of the time (12 years out of 24 years).

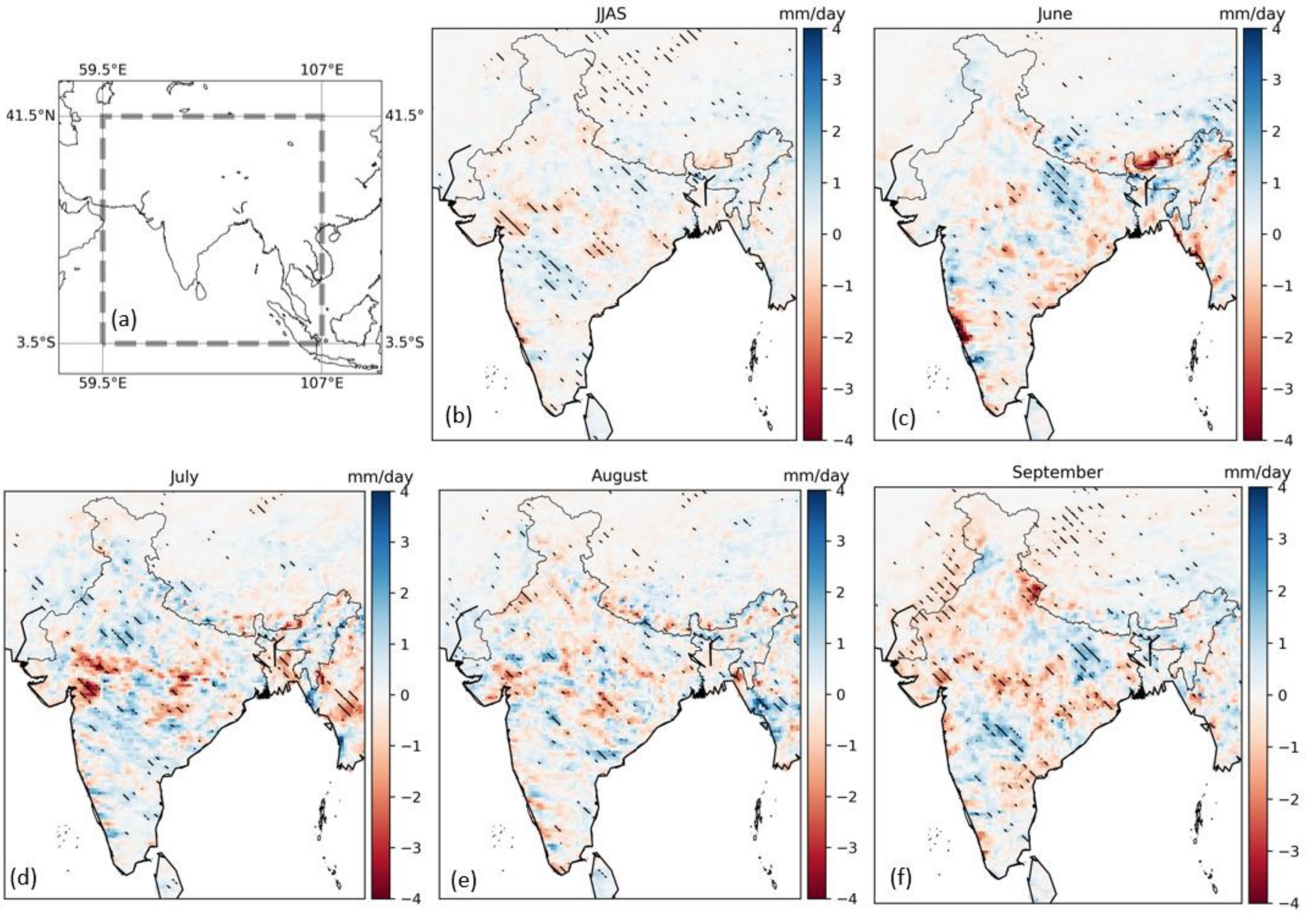

S5: (a) Domain used for WRF simulations. (b-e) The difference in mean Precipitation (mm/day) between irrigation run (IRR) and control run (CTL) runs from regional climate model simulations (WRF-CLM4) for the complete monsoon season (b, JJAS; June to September) and individual months June (c), July (d), August (e), September (f). Hatched regions indicate statistically significant differences at 90% confidence ($p<0.1$) tested on 660 data points.

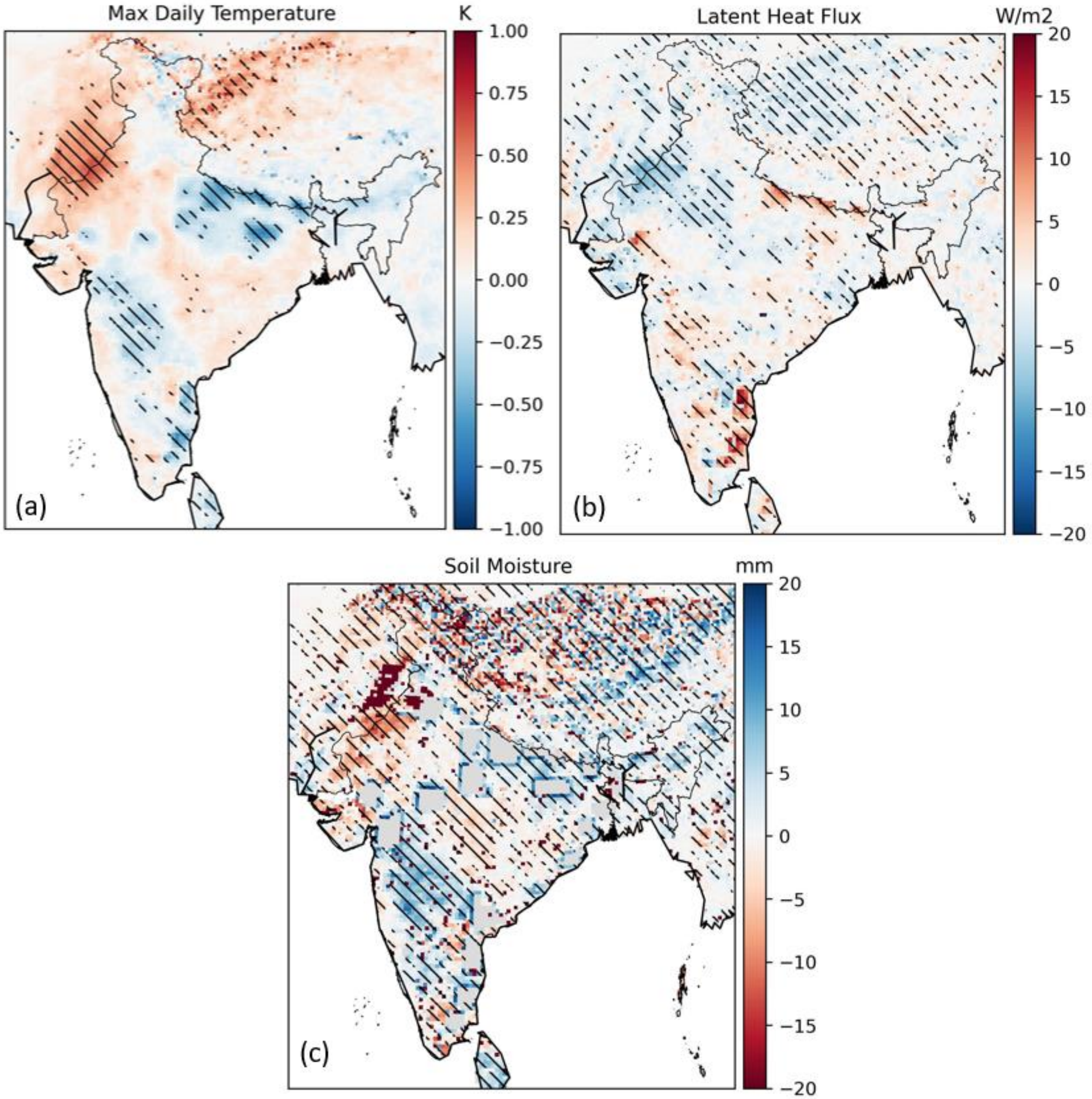

S6: (a-c) Differences between mean values from irrigation run (IRR) and control run (CTL) runs from the regional climate model simulations (WRF-CLM4) during the month of September for variables daily maximum temperature (a; K), mean daily latent heat flux (b; W/m2) and mean monthly soil moisture (c; mm). Hatched regions represent statistically significant grids at 90% confidence ($p<0.1$) tested on 660 data points.

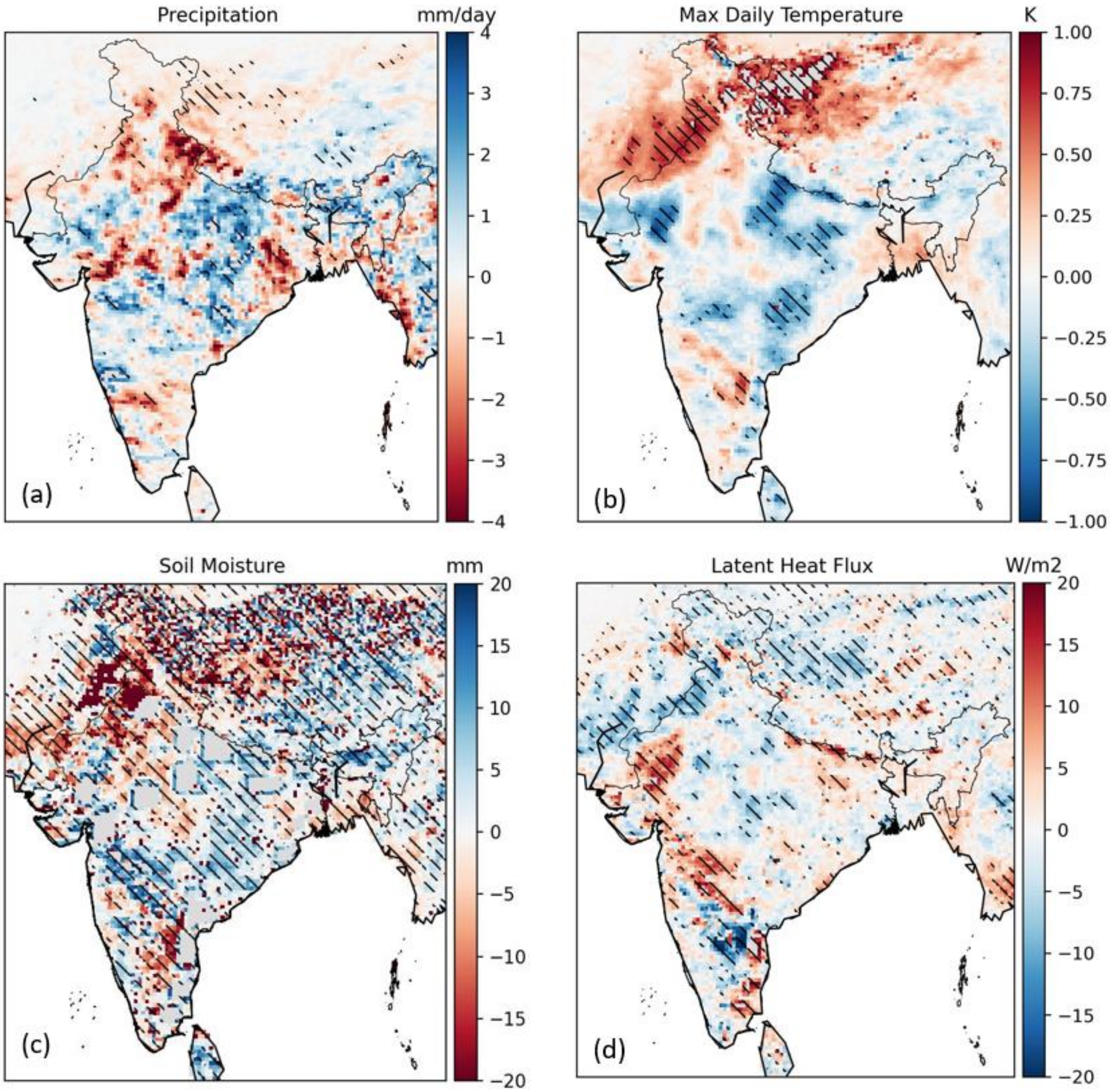

S7: Difference between mean values from irrigation run (IRR) and control run (CTL) runs from regional climate model simulations (WRF-CLM4) during September months of El Niño Years for daily precipitation (a; mm/day) , daily maximum temperature (b; K), mean monthly soil moisture (c; mm) and mean daily latent heat flux (d; W/m2). Hatched regions represent statistically significant grids at 90% confidence (p<0.1) tested on 150 data points.

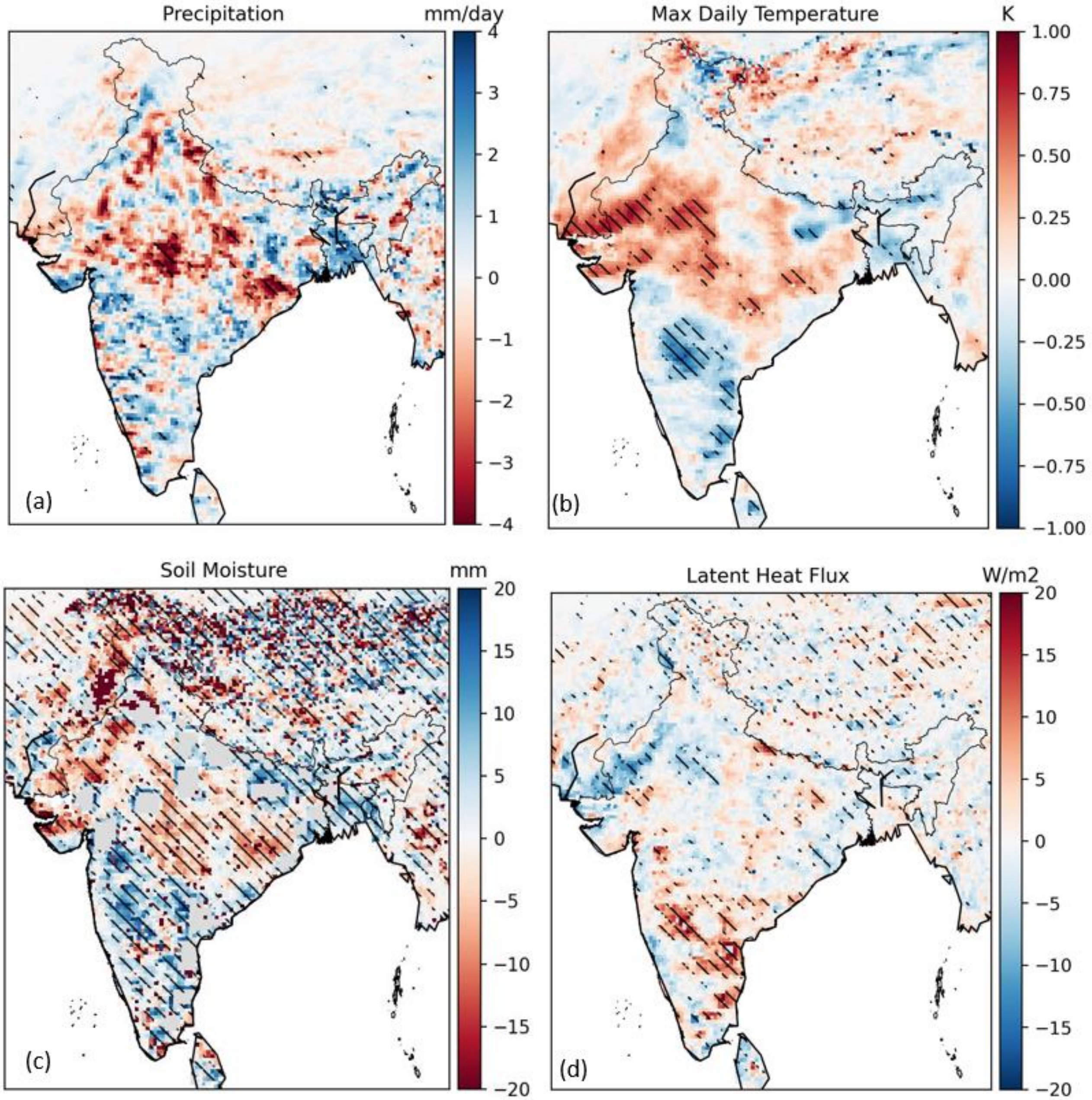

S8: Difference between mean values from irrigation run (IRR) and control run (CTL) runs from regional climate model simulations (WRF-CLM4) during September months of La Niña Years for daily precipitation (a, mm/day), daily maximum temperature (b, K), mean monthly soil moisture (c, mm),and mean daily latent heat flux (d, W/m2). Hatched regions represent statistically significant grids at 90% confidence (p<0.1) tested on 150 data points.

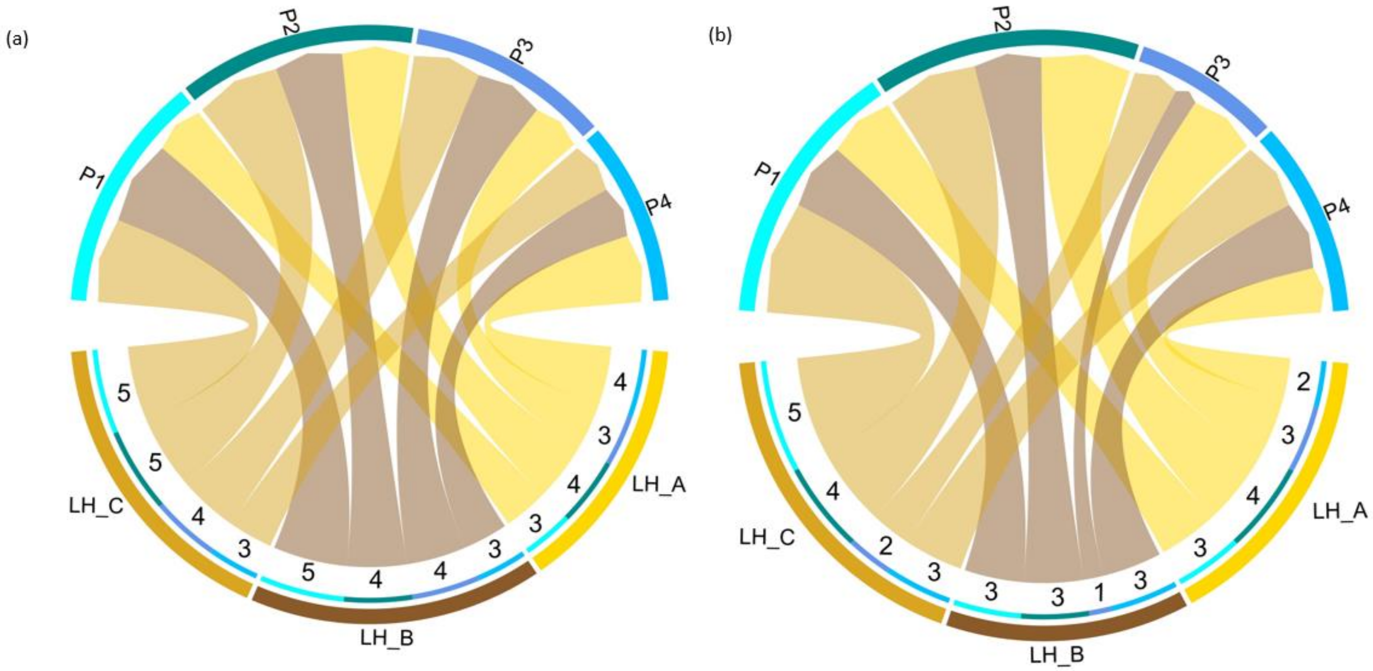

S9: Transfer Entropy (TE) form changes in irrigation run (IRR) to control run (CTL) (IRR-CTL) from latent heat flux of irrigated regions to precipitation change (IRR-CTL) in drying regions for El Niño years (a) and La Niña years (b). Links are labelled as the number of years, when they were found out to be statistically significant out of 5 El Niño and 5 La Niña years (ST 2). This shows the consistency of land-atmosphere feedback during both El Niño and La Niña years.

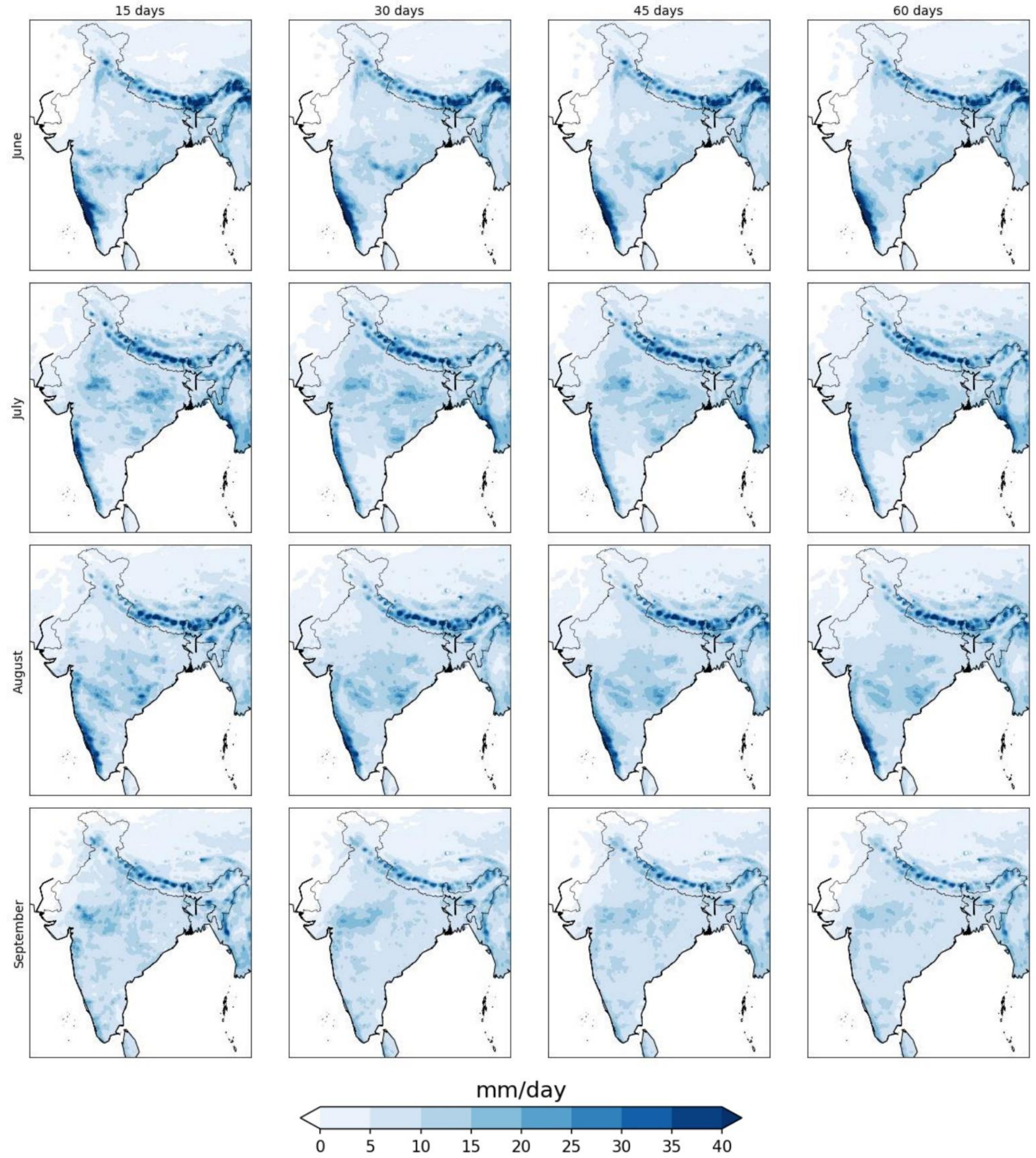

S10: Precipitation from regional climate model simulations (WRF-CLM4) control runs (CTL) for the year 2000 with varying spin-ups of 15, 30, 45, and 60 days. A spin-up of 60(45) days means WRF-CTL run was initialized from 1st (15th) April 2000.

Supplementary Tables

ST 1: Variables considered over each basin.

Data	Symbol	Spatial Resolution	Source
Precipitation	P	0.25° X 0.25°/0.5° X 0.667°	ERA-5/MERRA-2
Soil Moisture	SM	0.25° X 0.25°/0.5° X 0.667°	ERA-5/MERRA-2
Latent heat flux from land	LH	0.25° X 0.25°/0.5° X 0.667°	ERA-5/MERRA-2
Sensible heat flux from land	SH	0.25° X 0.25°/0.5° X 0.667°	ERA-5/MERRA-2
Shortwave flux incoming on land	SR	0.25° X 0.25°/0.5° X 0.667°	ERA-5/MERRA-2
Relative Humidity at 850 hpa	Q	0.25° X 0.25°/0.5° X 0.667°	ERA-5/MERRA-2
U-Wind at 850 hpa	U	0.25° X 0.25°/0.5° X 0.667°	ERA-5/MERRA-2
V-Wind at 850 hpa	V	0.25° X 0.25°/0.5° X 0.667°	ERA-5/MERRA-2
2 meter air Temperature	T	0.25° X 0.25°/0.5° X 0.667°	ERA-5/MERRA-2

ST 2: Years when Oceanic Ni**ñ**o index (ONI) exceeded 1 (El Ni**ñ**o) and was less than 1 (La Ni**ñ**a).

**El Niño**	**La Niña**
1991	1995
1994	1998
1997	1999
2002	2007
2009	2010

ST 3: Details of strengths and p-values of links in network generated of land variables using linear estimator of PCMCI. All values are statistically significant at more than 99% confidence.

Source	Target	Value	pValue	Source	Target	Value	pValue
LH_G	SH_G	0.040	<0.001	LH_K	LH_Go	0.075	<0.001
LH_G	SM_G	0.050	<0.001	LH_K	SH_Go	0.040	<0.001
LH_G	LH_Go	0.010	<0.001	LH_K	SM_Go	0.027	<0.001
LH_G	SH_Go	-0.001	<0.001	LH_K	LH_C	0.028	<0.001
LH_G	SM_Go	-0.004	<0.001	LH_K	SH_C	0.024	<0.001
LH_G	LH_C	-0.003	<0.001	LH_K	SM_C	0.010	<0.001
LH_G	SH_C	-0.005	<0.001	LH_K	SH_K	0.025	<0.001
LH_G	SM_C	-0.002	0.002	LH_K	SM_K	0.032	<0.001
LH_G	SM_K	0.002	0.007	LH_K	LH_M	0.027	<0.001
LH_G	LH_M	0.040	<0.001	LH_K	SH_M	0.013	<0.001
LH_G	SH_M	0.015	<0.001	LH_K	SM_M	0.007	<0.001
LH_G	SM_M	0.022	<0.001	LH_K	LH_NT	0.010	<0.001
LH_G	SH_NT	-0.005	<0.001	LH_K	SH_NT	0.001	<0.001
LH_G	SM_NT	-0.009	<0.001	LH_K	SM_NT	0.001	<0.001
SH_G	LH_G	0.042	<0.001	SH_K	SH_G	0.009	<0.001
SH_G	SM_G	0.052	<0.001	SH_K	SM_G	0.0001	0.001
SH_G	LH_Go	0.003	<0.001	SH_K	LH_Go	0.044	<0.001
SH_G	SH_Go	0.013	<0.001	SH_K	SH_Go	0.073	<0.001
SH_G	SM_Go	0.010	<0.001	SH_K	SM_Go	0.052	<0.001
SH_G	SH_C	-0.003	<0.001	SH_K	LH_C	0.017	<0.001
SH_G	SH_K	0.002	<0.001	SH_K	SH_C	0.035	<0.001
SH_G	SM_K	0.003	<0.001	SH_K	SM_C	0.014	<0.001
SH_G	LH_M	0.018	<0.001	SH_K	LH_K	0.026	<0.001
SH_G	SH_M	0.041	<0.001	SH_K	SM_K	0.062	<0.001
SH_G	SM_M	0.032	<0.001	SH_K	LH_M	0.022	<0.001
SH_G	LH_NT	-0.003	<0.001	SH_K	SH_M	0.030	<0.001
SM_G	LH_G	0.043	<0.001	SH_K	SM_M	0.021	<0.001
SM_G	SH_G	0.065	<0.001	SH_K	LH_NT	0.009	<0.001
SM_G	LH_Go	0.009	<0.001	SH_K	SH_NT	0.012	<0.001
SM_G	SH_Go	0.006	<0.001	SH_K	SM_NT	0.002	<0.001
SM_G	SM_Go	0.012	<0.001	SM_K	LH_G	-0.002	0.007
SM_G	LH_C	-0.002	0.001	SM_K	SH_G	0.002	<0.001
SM_G	SM_C	-0.002	0.010	SM_K	SM_G	0.002	0.002
SM_G	SH_K	0.005	0.001	SM_K	LH_Go	0.029	<0.001
SM_G	SM_K	0.002	0.002	SM_K	SH_Go	0.039	<0.001
SM_G	LH_M	0.020	<0.001	SM_K	SM_Go	0.065	<0.001
SM_G	SH_M	0.024	<0.001	SM_K	LH_C	0.021	<0.001

SM_G	SM_M	0.050	<0.001	SM_K	SH_C	0.030	<0.001
SM_G	LH_NT	-0.001	<0.001	SM_K	SM_C	0.024	<0.001
SM_G	SH_NT	-0.001	<0.001	SM_K	LH_K	0.032	<0.001
LH_Go	LH_G	0.009	<0.001	SM_K	SH_K	0.077	<0.001
LH_Go	SH_G	-0.0002	<0.001	SM_K	LH_M	0.003	<0.001
LH_Go	SM_G	0.0002	<0.001	SM_K	SH_M	0.010	<0.001
LH_Go	SH_Go	0.035	<0.001	SM_K	SM_M	0.023	<0.001
LH_Go	SM_Go	0.028	<0.001	SM_K	SH_NT	-0.0002	<0.001
LH_Go	LH_C	0.005	<0.001	SM_K	SM_NT	0.003	<0.001
LH_Go	SH_C	0.008	<0.001	LH_M	LH_G	0.043	<0.001
LH_Go	LH_K	0.065	<0.001	LH_M	SH_G	0.015	<0.001
LH_Go	SH_K	0.029	<0.001	LH_M	SM_G	0.022	<0.001
LH_Go	SM_K	0.020	<0.001	LH_M	LH_Go	0.057	<0.001
LH_Go	LH_M	0.044	<0.001	LH_M	SH_Go	0.039	<0.001
LH_Go	SH_M	0.021	<0.001	LH_M	SM_Go	0.026	<0.001
LH_Go	SM_M	0.009	<0.001	LH_M	LH_C	0.004	0.003
LH_Go	LH_NT	0.043	<0.001	LH_M	SH_C	0.003	<0.001
LH_Go	SH_NT	0.017	<0.001	LH_M	SM_C	-0.003	<0.001
LH_Go	SM_NT	0.015	<0.001	LH_M	LH_K	0.022	<0.001
SH_Go	LH_G	0.002	<0.001	LH_M	SH_K	0.025	<0.001
SH_Go	SH_G	0.016	<0.001	LH_M	SM_K	0.009	<0.001
SH_Go	SM_G	0.001	<0.001	LH_M	SH_M	0.039	<0.001
SH_Go	LH_Go	0.037	<0.001	LH_M	SM_M	0.045	<0.001
SH_Go	SM_Go	0.053	<0.001	LH_M	SH_NT	-0.0005	<0.001
SH_Go	LH_C	0.004	<0.001	LH_M	SM_NT	-0.003	<0.001
SH_Go	SH_C	0.013	<0.001	SH_M	LH_G	0.025	<0.001
SH_Go	SM_C	0.002	0.004	SH_M	SH_G	0.047	<0.001
SH_Go	LH_K	0.031	<0.001	SH_M	SM_G	0.017	<0.001
SH_Go	SH_K	0.065	<0.001	SH_M	LH_Go	0.027	<0.001
SH_Go	SM_K	0.047	<0.001	SH_M	SH_Go	0.057	<0.001
SH_Go	LH_M	0.028	<0.001	SH_M	SM_Go	0.034	<0.001
SH_Go	SH_M	0.052	<0.001	SH_M	SH_C	0.002	0.001
SH_Go	SM_M	0.030	<0.001	SH_M	LH_K	0.002	<0.001
SH_Go	LH_NT	0.016	<0.001	SH_M	SH_K	0.027	<0.001
SH_Go	SH_NT	0.033	<0.001	SH_M	SM_K	0.010	<0.001
SH_Go	SM_NT	0.016	<0.001	SH_M	LH_M	0.039	<0.001
SM_Go	LH_G	0.004	<0.001	SH_M	SM_M	0.055	<0.001
SM_Go	SH_G	0.013	<0.001	SH_M	SM_NT	-0.006	0.01
SM_Go	SM_G	0.012	<0.001	SM_M	LH_G	0.019	<0.001
SM_Go	LH_Go	0.042	<0.001	SM_M	SH_G	0.029	<0.001
SM_Go	SH_Go	0.071	<0.001	SM_M	SM_G	0.043	<0.001

SM_Go	LH_C	0.008	<0.001	SM_M	LH_Go	0.035	<0.001
SM_Go	SH_C	0.019	<0.001	SM_M	SH_Go	0.047	<0.001
SM_Go	SM_C	0.010	<0.001	SM_M	SM_Go	0.059	<0.001
SM_Go	LH_K	0.026	<0.001	SM_M	LH_C	0.003	<0.001
SM_Go	SH_K	0.050	<0.001	SM_M	SH_C	0.011	<0.001
SM_Go	SM_K	0.070	<0.001	SM_M	LH_K	0.002	<0.001
SM_Go	LH_M	0.017	<0.001	SM_M	SH_K	0.033	<0.001
SM_Go	SH_M	0.030	<0.001	SM_M	SM_K	0.027	<0.001
SM_Go	SM_M	0.045	<0.001	SM_M	LH_M	0.041	<0.001
SM_Go	LH_NT	0.009	<0.001	SM_M	SH_M	0.067	<0.001
SM_Go	SH_NT	0.013	<0.001	SM_M	LH_NT	-0.002	<0.001
SM_Go	SM_NT	0.034	<0.001	SM_M	SH_NT	-0.004	<0.001
LH_C	LH_G	-0.003	<0.001	SM_M	SM_NT	-0.010	<0.001
LH_C	SM_G	-0.0002	<0.001	LH_NT	SH_G	-0.009	<0.001
LH_C	LH_Go	0.012	<0.001	LH_NT	SM_G	-0.008	<0.001
LH_C	SH_Go	0.014	<0.001	LH_NT	LH_Go	0.037	<0.001
LH_C	SM_Go	0.008	<0.001	LH_NT	SH_Go	0.016	<0.001
LH_C	SH_C	0.043	<0.001	LH_NT	SM_Go	0.010	<0.001
LH_C	SM_C	0.042	<0.001	LH_NT	SM_C	-0.002	<0.001
LH_C	LH_K	0.034	<0.001	LH_NT	LH_K	0.010	<0.001
LH_C	SH_K	0.019	<0.001	LH_NT	SH_K	0.005	<0.001
LH_C	SM_K	0.018	<0.001	LH_NT	SM_M	-0.010	<0.001
LH_C	LH_M	0.002	0.003	LH_NT	SH_NT	0.025	<0.001
LH_C	SM_M	0.003	<0.001	LH_NT	SM_NT	0.036	<0.001
LH_C	SH_NT	0.002	0.006	SH_NT	LH_G	0.001	<0.001
SH_C	LH_G	-0.007	<0.001	SH_NT	SM_G	-0.013	<0.001
SH_C	SH_G	-0.003	<0.001	SH_NT	LH_Go	0.018	<0.001
SH_C	LH_Go	0.020	<0.001	SH_NT	SH_Go	0.046	<0.001
SH_C	SH_Go	0.022	<0.001	SH_NT	SM_Go	0.023	<0.001
SH_C	SM_Go	0.016	<0.001	SH_NT	LH_C	0.005	0.006
SH_C	LH_C	0.045	<0.001	SH_NT	LH_K	0.005	<0.001
SH_C	SM_C	0.056	<0.001	SH_NT	SH_K	0.010	<0.001
SH_C	LH_K	0.032	<0.001	SH_NT	SM_K	0.004	<0.001
SH_C	SH_K	0.039	<0.001	SH_NT	LH_M	-0.003	<0.001
SH_C	SM_K	0.025	<0.001	SH_NT	SM_M	-0.004	<0.001
SH_C	LH_M	0.005	<0.001	SH_NT	LH_NT	0.028	<0.001
SH_C	SH_M	0.005	0.001	SH_NT	SM_NT	0.061	<0.001
SH_C	SM_M	0.007	<0.001	SM_NT	LH_G	-0.003	<0.001
SM_C	LH_G	-0.002	0.002	SM_NT	LH_Go	0.011	<0.001
SM_C	SM_G	-0.002	0.010	SM_NT	SH_Go	0.013	<0.001
SM_C	SH_Go	0.002	0.004	SM_NT	SM_Go	0.024	<0.001

SM_C	SM_Go	0.003	<0.001	SM_NT	LH_K	-0.003	<0.001
SM_C	LH_C	0.042	<0.001	SM_NT	SH_K	-0.006	<0.001
SM_C	SH_C	0.067	<0.001	SM_NT	SM_K	0.000	<0.001
SM_C	LH_K	0.005	<0.001	SM_NT	LH_M	-0.003	<0.001
SM_C	SH_K	0.016	<0.001	SM_NT	SH_M	-0.002	0.009
SM_C	SM_K	0.032	<0.001	SM_NT	SM_M	0.002	<0.001
SM_C	LH_M	-0.008	<0.001	SM_NT	LH_NT	0.031	<0.001
SM_C	LH_NT	-0.007	0.005	SM_NT	SH_NT	0.051	<0.001
